# Supplementary material for: Correlates of SARS-CoV-2 Variants on Deaths, Case Incidence and Case Fatality Ratio among the Continents for the Period of 1 December 2020 to 15 March 2021
Source: Genes (Basel). 2021 Jul 12;12(7):1061. doi: 10.3390/genes12071061 (PMC8306878; doi:10.3390/genes12071061)
Supplement: Supplementary file 1 [file genes-12-01061-s001.zip › genes-1244710-supplementary.pdf]

Supplementary Materials:

Table S1: Clades frequencies of SARS-CoV-2 genome

Continent	SARS-CoV-2 clades frequencies						
	G	GH	GR	GV	L	S	V
Africa	44.56%	25.68%	28.09%	0.01%	0%	1.6%	0.04%
Asia	52.25%	7.75%	39.35%	0.02%	0%	0.61%	0.03%
Australia	48.94%	23.06%	26.23%	0.04%	0.08%	1.18%	0.48%
Europe	57.24%	4.59%	37.98%	0.01%	0.01%	0.14%	0.03%
North America	59.42%	31.24%	9%	0.03%	0%	0.29%	0.03%
South America	73.53%	4.32%	14.96%	4.48%	0.08%	0.77%	1.87%

Table S2: Correlation analysis between SARS-CoV-2 mutations and Case fatality rates, cases per million, and deaths per million among the identified mutations in all world continents.

**SARS-CoV-2 mutations**	Case fatality ratio		Deaths/ 1M pop		Cases/ 1M pop		SARS-CoV-2 mutations	Case fatality ratio		Deaths/ 1M pop		Cases/ 1M pop	
	r	p	r	p	r	p		r	p	r	p	r	p
**NSP2_T85I**	-0.029	0.957	-0.486	0.329	-0.543	0.266	NSP2_M135T	0.698	0.123	0.030	0.954	-0.213	0.686
NSP2_G339S	0.771	0.072	0.771	0.072	0.600	0.208	Spike_A1174V	0.696	0.125	0.087	0.870	-0.174	0.742
NSP5_A266V	-0.257	0.623	-0.543	0.266	-0.429	0.397	NSP2_V198I	-0.429	0.397	-0.714	0.111	-0.600	0.208
NSP12_P323L	0.086	0.872	0.886*	0.019	0.943**	0.005	NSP6_M86I	0.314	0.544	-0.371	0.468	-0.543	0.266
Spike_D614G	0.371	0.468	0.943**	0.005	0.886*	0.019	NSP13_H164Y	-0.771	0.072	-0.086	0.872	0.086	0.872
NS3_I35T	0.464	0.354	-0.145	0.784	-0.406	0.425	NSP5_G71S	0.6	0.208	0.486	0.329	0.429	0.397
Spike_D1139Y	0.135	0.798	0.541	0.268	0.541	0.268	NSP2_R380H	0.754	0.084	0.145	0.784	-0.116	0.827
N_G204R	-0.086	0.872	0.371	0.468	0.429	0.397	NSP2_I491T	0.000	1.000	0.696	0.125	0.696	0.125
N_R203K	-0.086	0.872	0.371	0.468	0.429	0.397	NSP3_P1326S	0.754	0.084	0.145	0.784	-0.116	0.827
NS3_S171L	0.086	0.872	-0.714	0.111	-0.771	0.072	NSP2_I296V	0.580	0.228	0.261	0.618	0.058	0.913
NSP6_L37F	-0.714	0.111	-0.714	0.111	-0.543	0.266	NSP2_T634A	0.135	0.798	0.541	0.268	0.541	0.268
N_S190I	-0.371	0.468	-0.600	0.208	-0.543	0.266	M_D3G	-0.086	0.872	0.714	0.111	0.771	0.072
NSP2_T153M	0.829*	0.042	0.257	0.623	-0.029	0.957	NSP3_P1558L	0.000	1.000	0.696	0.125	0.696	0.125
NSP2_V198I	-0.429	0.397	-0.714	0.111	-0.600	0.208	NSP16_V78G	-0.273	0.600	0.273	0.600	0.273	0.600
NSP3_E475D	-0.030	0.954	0.516	0.295	0.516	0.295	Spike_Y28H	-0.657	0.156	-0.086	0.872	0.200	0.704
NSP3_Q1884H	-0.429	0.397	-0.371	0.468	-0.257	0.623	NSP3_V1768G	-0.439	0.383	-0.034	0.949	-0.034	0.949
NSP3_T73I	-0.886	0.019	-0.314	0.544	-0.029	0.957	N_S202N	0.086	0.872	-0.657	0.156	-0.771	0.072
Spike_A871S	0.522	0.288	-0.087	0.870	-0.348	0.499	NS8_L84S	-0.143	0.787	-0.943**	0.005	-1.000**	0.005
NSP3_T1198K	-0.600	0.208	-0.886 *	0.019	-0.771	0.072	NSP3_T64P	0.393	0.441	0.655	0.158	0.655	0.158
NSP12_G228C	-0.152	0.774	0.395	0.439	0.395	0.439	N_S194L	-0.543	0.266	-0.429	0.397	-0.371	0.468
Spike_A958S	0.030	0.954	0.577	0.231	0.577	0.231	NSP12_A97V	-0.657	0.156	-0.943**	0.005	-0.829*	0.042
NSP2_S122	0.086	0.872	0.371	0.468	0.257	0.623	NSP15_V22L	0.371	0.468	0.257	0.623	0.200	0.704
NSP14_I42V	0.845*	0.034	0.068	0.899	-0.169	0.749	NS3_Q57H	-0.143	0.787	-0.600	0.208	-0.657	0.156
N_P344S	0.543	0.266	0.543	0.266	0.371	0.468	N_S197L	-0.600	0.208	0.029	0.957	0.257	0.623
NSP1_M85I	0.030	0.954	0.577	0.231	0.577	0.231	NS3_G251V	-0.086	0.872	-0.314	0.544	-0.257	0.623
NSP3_Q180H	-0.829*	0.042	-0.200	0.704	0.029	0.957	NSP2_G212D	-0.486	0.329	0.086	0.872	0.371	0.468
Spike_L18F	0.829*	0.042	0.143	0.787	-0.029	0.957	NSP3_A58T	-0.771	0.072	-0.200	0.704	0.086	0.872
Spike_E484K	0.257	0.623	-0.486	0.329	-0.600	0.208	Spike_N439K	-0.086	0.872	-0.029	0.957	0.086	0.872
Spike_H69del	0.086	0.872	-0.143	0.787	-0.086	0.872	Spike_A222V	0.257	0.623	0.029	0.957	0.086	0.872
Spike_K417N	0.200	0.704	-0.600	0.208	-0.657	0.156	Spike_S477	0.143	0.787	-0.429	0.397	-0.371	0.468
Spike_N501Y	0.543	0.266	0.086	0.872	0.029	0.957	Spike_T20N	-0.314	0.544	0.314	0.544	0.543	0.266

Spike_G476S	0.270	0.604	0.676	0.140	0.676	0.140	Spike_V483A	0.091	0.864	0.638	0.173	0.638	0.173
Spike_S477N	0.143	0.787	-0.429	0.397	-0.371	0.468	Spike_E484Q	0.464	0.354	-0.145	0.787	-0.406	0.425
Spike_T478I	0.290	0.577	-0.029	0.957	-0.232	0.658	Spike_Y453F	0.577	0.231	0.516	0.295	0.395	0.439
Spike_V1176F	-0.600	0.208	0.029	0.957	0.257	0.623	N_P13L	-0.714	0.111	-0.429	0.397	-0.200	0.704
Spike_E780Q	-0.429	0.397	-0.371	0.468	-0.257	0.623	NS3_V13L	-0.029	0.957	-0.314	0.544	-0.200	0.704
Spike_Y145del	-0.257	0.623	0.086	0.872	0.257	0.623	NS8_Q27stop	0.086	0.872	-0.143	0.787	-0.086	0.872
N_I292T	0.429	0.397	0.029	0.957	-0.086	0.872	NSP2_I559V	-0.143	0.787	-0.429	0.397	-0.314	0.544
NS8_S24L	-0.714	0.111	-0.086	0.872	0.143	0.787	NSP5_G15S	0.429	0.397	-0.029	0.957	-0.086	0.872
NSP2_D268del	-0.600	0.208	-0.314	0.544	-0.086	0.872	NSP13_Y541C	-0.829*	0.042	-0.257	0.623	0.029	0.957
NSP2_P585S	0.086	0.872	-0.486	0.329	-0.429	0.397	NSP13_P504L	-0.429	0.397	-0.143	0.787	0.086	0.872

*. Correlation is significant at the 0.05 level (2-tailed). **. Correlation is significant at the 0.01 level (2-tailed). r: Spearman's correlation. P: P-value.

Table S3: Dendrogram showing clustering of SARS-CoV-2 isolates and heatmap of the frequency of mutations of the SARS-CoV-2 genome by continent.

Dendrogram	Region	Virus isolates	NSP2_T85I	NSP2_G339S	NSP5_A266V	NSP12_P323L	Spike_D614G	NS3_I35T	Spike_D1139Y	N_G204R	N_R203K	NS3_S171L	NSP6_L37F	N_S190I	NSP2_T153M	NSP2_V198I	NSP3_E475D	NSP3_Q1884H	NSP3_T73I	Spike_A871S	NSP3_T1198K	NSP12_G228C	Spike_A958S	NSP2_S122	NSP14_I42V	N_P344S	NSP1_M85I	NSP2_M135T	Spike_A1174V	NSP2_V198I	NSP6_M86I	NSP13_H164Y	NSP5_G71S	NSP2_R380H	NSP2_I491T	NSP3_P1326S	NSP2_I296V	NSP2_T634A	M_D3G	NSP3_P1558L	NSP16_V78G	Spike_Y28H	NSP3_V1768G
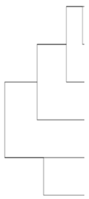	Asia	34,736	6.85	0.32	0.003	95.52	96.46	0.04	0.000	75.69	75.88	1.50	6.61	0.03	0.02	0.22	0.003	2.41	0.05	0.02	0.41	0.032	0.003	0.10	0.000	0.02	0.01	0.000	0.02	0.22	0.83	0.13	0.07	0.00	0.009	0.01	0.00	0.000	0.07	0.066	0.026	0.01	0.02
	South America	6,702	7.92	1.42	0.015	96.43	97.02	0.00	0.000	76.01	76.41	1.19	3.01	0.01	0.07	0.01	0.000	0.06	0.03	0.00	0.07	0.000	0.000	0.06	0.000	0.06	0.00	0.000	0.00	0.01	0.01	0.10	0.82	0.01	0.015	0.00	0.01	0.000	0.30	0.134	0.000	0.01	0.00
	Australia	1,670	24.97	0.12	0.838	91.08	91.02	0.00	0.000	46.59	47.90	3.29	7.37	0.12	0.00	0.66	0.000	0.24	0.18	0.00	1.56	0.000	0.000	0.00	0.000	0.00	0.00	0.000	0.00	0.66	0.18	0.12	0.18	0.00	0.000	0.00	0.00	0.000	0.06	0.000	0.000	0.06	0.00
	Europe	346,951	1.34	0.60	0.012	98.35	98.99	0.03	0.005	52.36	53.51	1.20	2.64	0.02	0.04	0.02	0.014	0.12	0.05	0.02	0.01	0.007	0.006	0.07	0.003	0.03	0.01	0.001	0.04	0.02	0.31	0.10	0.56	0.01	0.005	0.03	0.01	0.000	0.25	0.009	0.001	0.01	0.00
	Africa	5,768	40.20	0.40	0.035	88.66	91.97	0.14	0.000	22.31	24.24	30.37	2.98	0.07	1.18	0.10	0.000	0.09	0.02	0.05	0.21	0.000	0.000	0.07	0.017	0.03	0.00	0.017	0.09	0.10	5.27	0.07	0.36	0.05	0.000	0.03	0.02	0.000	0.00	0.000	0.000	0.00	0.00
	North America	157,691	59.59	0.33	0.068	94.79	97.91	0.07	0.005	14.82	15.08	0.72	2.88	1.36	0.07	0.01	0.031	0.04	0.10	0.01	0.02	0.003	0.003	0.13	0.000	0.04	0.01	0.001	0.02	0.01	0.08	0.13	0.08	0.03	0.002	0.02	0.08	0.001	0.63	0.001	0.015	0.05	0.01

Dendrogram	Region	Virus isolates	N_S202N	NS8_L84S	NSP3_T64P	N_S194L	NSP12_A97V	NSP15_V22L	NS3_Q57H	N_S197L	NS3_G251V	NSP2_G212D	NSP3_A58T	NSP3_Q180H	Spike_L18F	Spike_E484K	Spike_H69del	Spike_K417N	Spike_N439K	Spike_N501Y	Spike_A222V	Spike_G476S	Spike_S477	Spike_S477N	Spike_T20N	Spike_T478I	Spike_V483A	Spike_V1176F	Spike_E484Q	Spike_E780Q	Spike_Y453F	Spike_Y145del	N_P13L	N_I292T	NS3_V13L	NS8_S24L	NS8_Q27stop	NSP2_D268del	NSP2_I559V	NSP2_P585S	NSP5_G15S	NSP13_P504L	NSP13_Y541C
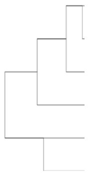	Asia	34,736	1.01	1.39	0.000	7.94	0.52	0.02	15.21	0.04	0.11	0.01	0.09	0.15	0.72	0.90	3.69	0.22	0.61	4.07	0.78	0.00	0.33	0.29	0.03	0.27	0.003	0.47	0.02	0.01	0.00	0.04	0.63	0.02	0.10	0.45	3.56	0.02	0.01	0.01	0.21	0.03	0.01
	South America	6,702	0.03	0.25	0.000	1.46	0.09	0.04	14.71	4.12	1.24	0.04	0.03	0.10	5.94	15.58	2.54	0.04	0.07	8.27	1.06	0.00	1.85	1.78	5.36	0.00	0.000	29.30	0.00	0.15	0.00	0.03	1.49	20.50	0.37	0.88	2.78	0.01	0.06	0.03	8.95	0.19	0.04
	Australia	1,670	0.48	3.29	0.000	6.71	1.98	0.06	32.99	1.80	1.08	0.36	0.36	0.18	1.86	3.11	12.69	2.34	1.86	14.31	2.75	0.00	16.23	16.23	0.12	0.00	0.000	0.90	0.00	0.18	0.00	0.06	3.29	0.06	0.48	4.73	13.35	0.18	0.54	0.60	1.56	0.84	0.84
	Europe	346,951	0.22	0.25	0.001	2.14	0.05	0.14	7.73	0.04	0.10	0.05	0.04	0.08	10.90	0.85	48.93	0.56	3.97	47.23	26.59	0.02	5.38	5.18	0.08	0.01	0.005	0.26	0.02	0.01	0.29	0.21	0.10	0.04	0.21	0.38	46.36	0.02	0.03	0.03	0.53	0.06	0.01
	Africa	5,768	4.09	4.09	0.000	3.66	0.28	0.10	46.10	0.02	0.16	0.00	0.00	0.05	16.02	35.63	4.20	34.36	0.10	36.55	1.37	0.00	5.39	5.36	0.00	0.03	0.000	0.10	0.03	0.09	0.02	0.03	0.42	0.54	0.24	0.17	3.81	0.02	0.03	0.03	2.98	0.03	0.00
	North America	157,691	0.11	0.51	0.000	14.28	0.23	0.34	63.30	0.23	0.06	0.01	0.01	1.18	1.25	1.79	3.48	0.07	0.06	3.57	0.50	0.01	0.94	0.83	0.03	0.01	0.004	0.53	0.03	0.80	0.02	0.05	0.96	0.13	0.17	33.88	3.31	0.06	0.03	0.03	0.29	0.39	0.15
